# Supplementary material for: Bright multicolor labeling of neuronal circuits with fluorescent proteins and chemical tags
Source: eLife. 2018 Nov 20;7:e40350. doi: 10.7554/eLife.40350 (PMC6245733; doi:10.7554/eLife.40350)
Supplement: Transparent reporting form [file elife-40350-transrepform.docx]

***eLife’s* transparent reporting form**

We encourage authors to provide detailed information *within their submission* to facilitate the interpretation and replication of experiments. Authors can upload supporting documentation to indicate the use of appropriate reporting guidelines for health-related research (see [EQUATOR Network](http://www.equator-network.org/%20)), life science research (see the [BioSharing Information Resource](https://biosharing.org/)), or the [ARRIVE guidelines](http://www.plosbiology.org/article/info:doi/10.1371/journal.pbio.1000412) for reporting work involving animal research. Where applicable, authors should refer to any relevant reporting standards documents in this form.

If you have any questions, please consult our Journal Policies and/or contact us: [editorial@elifesciences.org](mailto:editorial@elifesciences.org).

**Sample-size estimation**

- You should state whether an appropriate sample size was computed when the study was being designed
- You should state the statistical method of sample size computation and any required assumptions
- If no explicit power analysis was used, you should describe how you decided what sample (replicate) size (number) to use

Please outline where this information can be found within the submission (e.g., sections or figure legends), or explain why this information doesn’t apply to your submission:

No statistical methods were used to pre-determine the sample sizes. As the sample size is not in itself an important characteristic in our qualitative descriptions (Figs 4-6, and 8). However, for the testing of the quality of the constructs, at least 3 animals were used for each combination of Tetbow for *in utero* electroporation (Fig 2, and 3). And, at least 2 animals were used for each combination of Tetbow for AAV (Fig 7). Showing that it is indeed a reliable method (sample sizes are described in the figure legends).

For the quantitative comparison of intensities between some constructs (Figure 2E, 3B, 3E, and 7C), power analysis was performed for a One-Way ANOVA. For the comparison of percentage of discernable cells in the condition of each threshold Euclidean distance between some constructs (figure 2F, 3C, 3F, 7D), power analysis was performed for a MANOVA. power analysis for all analysis was conducted by G*power.

For Figure 2E, power = 0.9996754. For Figure 2F, power = 1.0000000.For Figure 3B, power = 1.0000000. For Figure 3C, power = 1.0000000. For Figure 3E, power = 1.0000000. For Figure 3F, power = 1.0000000. For Figure 7C, power = 0.9999085. For Figure 7D, power = 1.0000000.

The sample size for each analysis is indicated in the corresponding part of the main text, figure legends, and/or Methods section.

**Replicates**

- You should report how often each experiment was performed
- You should include a definition of biological versus technical replication
- The data obtained should be provided and sufficient information should be provided to indicate the number of independent biological and/or technical replicates
- If you encountered any outliers, you should describe how these were handled
- Criteria for exclusion/inclusion of data should be clearly stated
- High-throughput sequence data should be uploaded before submission, with a private link for reviewers provided (these are available from both GEO and ArrayExpress)

Please outline where this information can be found within the submission (e.g., sections or figure legends), or explain why this information doesn’t apply to your submission:

Image data were excluded from further quantitative analyses when the data contained saturated fluorescence signals. This is explained in Methods section. Sample sizes and accompanying statistics are in the figure legends and/or Methods section.

There were no technical replicates in any of the data sets used.

**Statistical reporting**

- Statistical analysis methods should be described and justified
- Raw data should be presented in figures whenever informative to do so (typically when N per group is less than 10)
- For each experiment, you should identify the statistical tests used, exact values of N, definitions of center, methods of multiple test correction, and dispersion and precision measures (e.g., mean, median, SD, SEM, confidence intervals; and, for the major substantive results, a measure of effect size (e.g., Pearson's r, Cohen's d)
- Report exact p-values wherever possible alongside the summary statistics and 95% confidence intervals. These should be reported for all key questions and not only when the p-value is less than 0.05.

Please outline where this information can be found within the submission (e.g., sections or figure legends), or explain why this information doesn’t apply to your submission:

The statistical test for each experiment is clearly defined in the Results section and/or figure legends.

For figure 2E, 3B, 3E, and 7C, non-parametric statistical analyses were performed as the data was not normally distributed (D’Agositino and Pearson Normality Test p<0.0001 for both intensity and spread measurements).

Other statistical values for the corresponding statistical tests are provided in Figure Legends.

(For large datasets, or papers with a very large number of statistical tests, you may upload a single table file with tests, Ns, etc., with reference to sections in the manuscript.)

**Group allocation**

- Indicate how samples were allocated into experimental groups (in the case of clinical studies, please specify allocation to treatment method); if randomization was used, please also state if restricted randomization was applied
- Indicate if masking was used during group allocation, data collection and/or data analysis

Please outline where this information can be found within the submission (e.g., sections or figure legends), or explain why this information doesn’t apply to your submission:

Image data was acquired by RS, to prevent bias they were subsequently analyzed by ML. This is explained in Methods section.

**Additional data files (“source data”)**

- We encourage you to upload relevant additional data files, such as numerical data that are represented as a graph in a figure, or as a summary table
- Where provided, these should be in the most useful format, and they can be uploaded as “Source data” files linked to a main figure or table
- Include model definition files including the full list of parameters used
- Include code used for data analysis (e.g., R, MatLab)
- Avoid stating that data files are “available upon request”

Please indicate the figures or tables for which source data files have been provided:

Raw numerical values are provided for Figure 1E, 1G, 1H, 2C, 2D, 2E, 3B, 3C, 3E, 3F, 7B, and 8E in source data.

Processed data for figure panels (e.g., normalized values, Euclidean distances) are provided for mat files.

Poisson Distributions.mat and Poisson Distributions_Analysed.mat includes data for figure 1E and G.

CAGvsTetBOW.mat includes data for figure 2C, D, and F.

XFPvariations.mat includes data for figure 3C.

TreVariations.mat includes data for figure 3F.

AAVbow.mat includes data for figure 7B and D.

MATLAB code and processed data for figure panels will be deposited to GitHub upon publication.

All raw images will be available from the Systems Science of

Biological Dynamics (SSBD) database upon publication.
